# Grandma's House: A Community‐Led Partnership With University Researchers Drawing on Cultural Heritage Approaches to Support Research Development

**DOI:** 10.1111/hex.70932

**Published:** 2026-10-27

**Authors:** Adele Horobin, Monica Tucker, Shanine Fasasi, Gemma Poole, Khadiyo Hassan, Insa Hussain, Joanne R. Morling

**Affiliations:** ^1^ School of Medicine, University of Nottingham Nottingham Nottinghamshire UK; ^2^ Public Contributor; ^3^ Diversify and Education CIC Nottingham Nottinghamshire UK; ^4^ The Essential Baby Company Nottingham Nottinghamshire UK

**Keywords:** African‐Caribbean, co‐design, co‐development, community engagement, research partnerships

## Abstract

**Background:**

In African‐Caribbean culture, the grandmother occupies a central moral and social position, a matriarch. In the context of community engagement, this imagery serves as a metaphor for relational and cultural intelligence in partnerships. At the suggestion of local community members, we sought to leverage this to form a community research partnership.

**Objective:**

We describe, critically review and outline the key learning points from the first year of delivering Grandma's House.

**Methods:**

The extended appreciative inquiry cycle (Define, Discover, Dream, Design and Destiny) was used to co‐produce Grandma's House. Each stage was explored through meetings and workshops with community members to support the delivery of pilot events. The approach was continuously evaluated through event feedback and a final broad stakeholder meeting, where a review and SWOT analysis were conducted.

**Results:**

Grandma's House successfully replicated the traditional African‐Caribbean generation‐to‐generation support model. Five pilot events were successfully delivered in a range of local community settings. Key strengths were identified as collective learning and knowledge sharing; active community engagement through heritage; and building community‐university relationships. Weaknesses included the challenge of funding and university inflexibility to align with community preferences.

**Discussion:**

We successfully implemented a unique approach to facilitate a critical shift in the role of community members in leading collaborations in health research and practice. Grandma's House worked because it came from the community, beginning with cultural pride rather than community need.

**Public Contribution:**

The public were the originators and drivers of this project, involved in every aspect and leading the majority. They supported the design, implementation, reflections and manuscript writing.

## 1 Background

### 1.1 Shifting the Focus From Public Involvement to Public Engagement

Patient and public involvement (PPI) in research projects was introduced in the NHS in 1996 as Consumers in NHS Research and became established as INVOLVE in 2003. Adopted by the NIHR at its formation in 2006 [1], it is now increasingly mandated by major research funders, including the NIHR and research‐active charities. The direction of travel is one where researchers must build strong links with diverse communities to enable successful funding bids, meaningful involvement and research which addresses the needs of people who have hitherto been underserved.

Support for continued PPI contact and input following project completion is sparse. Consequently, carefully nurtured relationships with our communities often fade, communities can feel let down, and our research community misses out on their input into further bids, projects and shared learning opportunities.

It is imperative that for research to continue flourishing, we must find better, more intelligent ways of working with our diverse, local communities. A platform is needed for communities and researchers to meet, learn from each other, build confidence to work together so that mutually beneficial agendas can be identified and carried forward. In short, researchers need to be supported to engage with local communities [2].

### 1.2 African‐Caribbean Communities Are Underserved in Research

All ethnic minority groups are impacted by inequalities in access to healthcare, research inclusion and public involvement in research opportunities. A recent report from the Partnership for Black People found that despite this, Black people want to have a voice, but that services and processes were hard to reach (as opposed to people being hard to reach) [3].

In the United Kingdom, only 1% of COVID‐19 vaccine trial participants were black despite making up 4% of the population [3]. Underrepresentation isn't unique to the United Kingdom, with a US‐based study revealing that black participants were underrepresented in 230 US‐based vaccine trials (2011–2020) at only 10.6% compared with 13.9% of the US adult population [4]. The inequity is not limited to vaccine trials, with systematic reviews demonstrating under‐representation of black people in both treatment and disease screening studies [5, 6].

One approach to improving research inclusion is to ensure appropriate and representative inclusion of diverse voices shaping the research. Yet the most recent UK surveys found only 2% of those involved in public involvement and engagement were Black [7]. Furthermore, it is known from other work that those from Black and other minority ethnic backgrounds have less confidence in the systems delivering research [8]. As more research is undertaken in the evaluation of health and care research, the context and background of participants will become even more important [9].

### 1.3 The Grandmother

In African‐Caribbean culture, the grandmother occupies a central moral and social position; a matriarch whose presence embodies continuity, resilience, and ancestral wisdom. Often described as the ‘heart of the home’, she anchors family and community life through acts of care, storytelling, and spiritual guidance. Her authority is rooted not in formal power but in lived experience: the quiet endurance of migration, the passing down of proverbs and remedies, the ritual of Sunday dinners that gather scattered generations. As a cultural custodian, she preserves traditions—from language and food to faith and folklore—that link descendants to their histories and homelands. Yet her influence extends beyond the domestic sphere; she is also a mediator, counsellor, and moral compass within the wider community, modelling compassion, discipline, and dignity. She is a vessel of social capital, sustaining collective identity and intergenerational trust [10, 11]. Her role illustrates how community knowledge often resides in lived practice rather than written record, and how the wisdom needed to heal, guide, and nurture already exists within the community itself, waiting to be recognised, respected, and supported [12, 13].

### 1.4 Grandma's House

If we consider the Grandmother through the lens of Appreciative Inquiry [14] and Asset‐Based Community Development [15], this challenges deficit‐based views of communities. By emphasising the resources, strengths, and wisdom already present within them, we can position the community as not requiring any external expertise.

In the context of community engagement, this imagery serves as a metaphor for relational and cultural intelligence in partnerships. It reinforces the argument that effective collaboration between researchers and communities depends on trust‐building [16] reciprocity [17], and the creation of collaborative advantage [18]. Like the grandmother who mediates relationships and nurtures collective wellbeing, community partners contribute forms of insight and relational labour that are vital to co‐produced research.

Gadamer's hermeneutic philosophy argues that understanding emerges through the interpretation of shared histories and traditions [19]. Grounding engagement in cultural heritage can lower barriers to participation by affirming local knowledge and lived experience, countering perceptions that engagement processes are externally imposed or technocratic [20, 21, 22]. From this perspective, heritage operates not merely as a communication tool but as a relational bridge, enabling co‐production through recognition and respect.

## 2 Aims and Objectives

Grandma's House was an idea envisaged by M.T. (a public contributor and key connector with local African‐Caribbean communities) and championed by M.T. and local community leaders (S.F., G.P.). Grandma's House will join these communities with researchers from across the School of Medicine, encouraging more open conversations, building trust and stimulating new ideas.

Sited within local ethnically diverse and socially deprived city wards, young and old will be invited to interact and learn from each other. Grandma's House will be a culturally appropriate vehicle for creating a positive impact, built around the needs of the people involved.

This paper describes the work conducted between the School of Medicine, University of Nottingham and local African‐Caribbean communities, and how we sought to build trusting and sustainable relationships by establishing a community‐led, shared initiative.

We describe, critically review and outline the key learning points from the first year of delivering Grandma's House.

## 3 Methods

### 3.1 Theoretical Framing

As described earlier, this project centres on asset‐based community development theory [6], using an appreciative inquiry framework [5] to support organisational development.

The appreciative inquiry cycle has been extended to include five stages: Define, Discover, Dream, Design and Destiny [23], which were used to co‐produce Grandma's House (Table 1).

**Table 1:** The 5D's of appreciative inquiry applied to Grandma's House.

5D's	Grandma's House
Define	Learning from each other about how they could build vibrant, healthy, strong partnerships between African‐Caribbean communities and researchers.
	Discovering new ways of collaborating with their partners as equals.
Discover	Utilise African‐Caribbean norms around the role of the Grandmother.
	Optimise the positives of intergenerational dialogue.
	Build on extensive existing community groups and connections.
Dream	Harness these to recreate intergenerational dialogue and celebrate the status and wisdom of the elders.
	Share and spread this wisdom with other generations.
	Normalise engagement between the community and researchers.
	Allow researchers to participate in and/or be party to, some of the lived experiences of the community.
	Create an environment where researchers can ‘drop in’ to discuss ideas with the community.
Design	Reproduce the cultural concept of Grandma for local communities and researchers through Grandma's House.
	Development of a structure that would allow Grandma's House to be iteratively co‐designed and delivered.
	To design the approach with the core principles of community first and giving to, not taking from, communities
	Flexible approach that is responsive to maximising opportunities as they are presented.
	No fixed location.
Destiny	Initial delivery of three pilot events.

### 3.2 Practical Development (Define, Discover, Dream)

An initial leadership team for Grandma's House was agreed consisting of community members (G.P., M.T., S.F.) and university members (A.H., J.R.M.) supported by Code of Conduct and Working Together (Terms of Reference) documents, which emphasised the approach of collaborative support and community leadership.

Decisions were made through discussion and consensus by the leadership team, holding to the agreed core principles of community first and giving to, not taking from, communities.

Across five development sessions, we moved continuously through the Define, Discover, Dream stages. Initially, discussion sessions were used to identify the ‘best’ aspects of what was already present. Considering questions such as ‘Tell me about a time when you felt at your best?’ Moving on to consider our aspirations using prompts such as ‘What would be the most exciting thing that could happen here?’ and ‘What can you take from your previous experiences that might be useful here?’.

We acknowledged one another's strengths and led from these positions. Community leaders led on defining Grandma's House, proposing activities to do (and with which communities), whether to include mention of research, pace of development and how to be culturally mindful in what we do and who is involved. University staff led on facilitating, co‐ordinating, identifying, obtaining and administering funding, and linking with relevant academic/clinical staff for events.

Approximately 50% of the funds achieved were paid directly to community organisations for their support in co‐developing Grandma's House, with the remainder used for the direct delivery costs associated with events. Community leaders guided the team on what activities to run, which venues to use, who else to involve, how to promote events and produced promotional material. Access to existing community networks was essential to delivering events, introducing key members of local communities as required.

The team decided on three initial events, which were agreed to include a range of venues and audiences, including a school, a church and a community centre, in order to help establish the deliverability of Grandma's House in different settings. A further stakeholder review event was added, and a contribution to a community retreat event following the emergence of additional opportunities from the initial events.

### 3.3 Review

We undertook an iterative, informal review of Grandma's House throughout its development.

We were keen to avoid an obtrusive or ‘clipboard’ approach to evaluating Grandma's House and wanted to find a way for the community to provide feedback with minimal burden or direction. At each event, participants were invited to provide their comments and reflections anonymously through written notes. To support accessibility, where necessary, thoughts were dictated onto paper. Through this, we could gauge whether our events were positively received, building our understanding of how well we were engaging. We applied this approach using small sticky notes to all our events except the school event, where we scribed comments from small table discussions between elders and students and gathered post‐event feedback from the link teacher. Given the exploratory and community‐development aims of the project, a formal qualitative analytic methodology was not employed. Instead, comments were reviewed iteratively by members of the project team, including community and academic partners, to identify recurring issues, reflections and areas of learning. Notes from event debriefs were compared with participant feedback to establish areas of convergence, which informed the themes presented in this paper.

Towards the end of the initial funding period, we were able to demonstrate sufficient engagement to be supported with additional funding to hold an in‐person stakeholder event. This afforded the opportunity for an in‐person evaluation activity.

### 3.4 Ethical Considerations

This work comprised public and patient involvement and community engagement activities conducted to inform project development and was therefore not subject to formal research ethics review. Specific approaches to ensure participant safety and appropriate use of data included:

− All those under the age of 18 years or meeting a vulnerability criterion were accompanied and supported at all times by a responsible adult known to them.
− All venues were risk assessed, and first aiders were present.
− The purpose of Grandma's House and its exploratory nature were introduced at the start of each session. Participants were made aware that they could leave at any time without reason—in line with local safeguarding processes.
− As part of the introduction, participants were made aware that any feedback or session contributions would be shared back with them and would be used as part of the ongoing development and learning for Grandma's House. Including potential publications.
− Participant contributions are reported in anonymised summary form.

## 4 Results

### 4.1 Grandma's House: The Vision (Destiny)

Guided by MT and community leaders (S.F., G.P.), we developed the concept of Grandma's House as a celebration of the wisdom, status, and cultural heritage of grandmothers. The vision was for Grandma's House to replicate this generation‐to‐generation support model. Working with community members, highlighting and applying the community's shared cultural heritage to strengthening community cohesion, creating educational opportunities, tackling taboos and building stronger relationships between community members, researchers and health and social care services. This is to be achieved through creating events that connect generations through shared learning, storytelling, and relationship‐building.

Through Grandma's House events, we also hoped to better understand how universities and researchers can engage with local African‐Caribbean communities – learning what health topics matter most to them and how to approach these in ways that are culturally respectful and relevant.

Ultimately researchers will be able to drop in to Grandma's House to:

- join activities, building mutual trust and confidence, removing one‐sided power dynamic weighted towards academia.
- understand the day‐to‐day health issues people face. Share their knowledge on health‐related topics.
- develop community‐centred ideas for research, co‐produced with the community.

### 4.2 Pilot Event Delivery (Design)

We successfully delivered five Grandma's House events in the first year of Grandma's House, of which the key characteristics (including costs in GBP as at December 2025) are described in Table 2.

**Table 2:** Grandma's House event characteristics.

Event	Setting	Key host organisation contributors	Number of Elders attending	Number of public attendees	Team attendees	Invited academics	Costs
Stephen Lawrence Day at Fernwood School	Secondary school. Yr10 (aged 14–15 years) children.	Lead teaching staff member.	4	15 pupils	5	0	£690 Taxis for Elders and time reimbursement through shopping vouchers.
International Children's Day at Pilgrim Church	Sunday church service. Congregation of all ages.	Senior Reverend.	2	~50	5	0	£850 Taxis for Elders and time reimbursement through shopping vouchers. Food costs. Shopping voucher donation to the church.
Windrush Celebration at Beechdale Community Centre	Community play and celebration event. Attended by local community members of all ages.	Diversify, Education and Communities CIC	0	~100	4	2	£1220 Taxis for Elders and time reimbursement through shopping vouchers. Food costs. Entertainment and other event costs.
Dads and Lads/StrongHER retreat	Hotel and grounds, bringing parents and young people together. Workshops around parenting, finance, personal development, careers and fun, team building exercises.	Diversify, Education and Communities CIC	6	40	2	0	£2405 Travel, subsistence and overnight accommodation costs for M.T. and attending Elders.
Grandma's House stakeholder event	University conference venue, bringing the team, elders, youth, academics, service providers, daycare service users for celebration, reflections and priority‐setting.	University of Nottingham	6	24	5	1	£2520 Venue hire and catering. Elder time reimbursement.

#### 4.2.1 Stephen Lawrence Day at Fernwood School, Nottingham

Community leaders linked with the school's Equality, Diversity and Inclusion Lead, who recommended the most appropriate way to bring the Elders of Grandma's House to meet with the children. They also guided on how best to link with the curriculum, the go/no go areas, inviting children and supporting facilitating the event.

The EDI Lead identified 15 Year 10 students and enabled their attendance at the event during school time in place of a usual class. Four Elders (accompanied by M.T.) joined the students, and the session was arranged as an Elder and Grandma's House team member at each of four tables with a small number of students. The school lead introduced the event covering themes around belonging and growing up in Jamaica or in England. This included questions to help prompt discussions. M.T. introduced the class to Grandma's House, and S.F. gave an introduction to the work Diversify, Education and Communities CIC delivers with local communities. Round table discussions ensued, and facilitators took notes on flip chart paper.

#### 4.2.2 International Children's Day at the Pilgrim Church

Community leaders linked with the senior pastor of a local church with a thriving, largely African‐Caribbean congregation. The Reverend subsequently joined with us in planning the event and gave invaluable guidance on how we should join in with the church service activities.

Supported by the church senior leadership team, our team attended the Pilgrim Church at its Sunday service, coinciding with International Children's Day celebrations. We welcomed two elders (one of whom had attended the earlier Fernwood School event) to join us at the service too. The Reverend led in welcoming us to the church at the service and introduced Grandma's House to the congregation. Following the service, M.T. and G.P. laid out community‐prepared African‐Caribbean food in an adjoining room, and the congregation were welcomed to join us and enjoy the food together. Discussions were prompted around the themes of the importance of food and family, and a board was set up for attendees to share their thoughts.

#### 4.2.3 Windrush Celebration at Beechdale Community Centre, Nottingham

The community partner Diversify, Education and Communities CIC (S.F.) is based at a local community centre and runs a well‐established calendar of events. In line with utilising existing community strengths, we were invited to conduct activities in parallel with a Windrush event. The community leaders proposed skin health as a focus for Grandma's House at the event.

Our team joined the Community Centre's presentation of a play based on Andrea Levy's Notes from a Small Island. The production integrated health themes and referenced how Windrush newcomers felt that the NHS was not meeting their needs upon their arrival in the United Kingdom. We held two tables at the back of the hall where people were seated for the play—one showcasing traditional skincare remedies as suggested by Elders and community partners, and the other around skin health, including eczema, recognising jaundice in babies with dark skin, and Vitamin D deficiency (hosted by a researcher and clinician from Centre for Academic Dermatology, University of Nottingham).

SF and MT introduced Grandma's House and our team members to the audience before the play. After the play, our Dermatology colleagues were on hand to provide expert advice, explain what they are doing to improve treating eczema in people with dark skin and to gather questions for future research addressing skin health in dark skin tones.

In this event, catering of authentic Caribbean cuisine was also provided for attendees, as well as a local musician and pastor who provided light entertainment.

#### 4.2.4 Dads and Lads, and StrongHER Retreat

Elders and MT joined with S.F. and her CIC team for Beechdale Community Centre's retreat weekend for their Dads and Lads and StrongHER groups to Crewe, adding value by involving the grandparent generation. Activities designed to nurture personal growth, encourage open conversations and intergenerational contact included fun activities such as the young dressing in 1940s and 1950s style and elders dressing in teenagers' apparel.It also included serious discussions about parenting and how it has changed through generations, business and entrepreneurship for young people, a youth breakout workshop to discuss drinking, vaping, smoking and leading a healthy lifestyle and an intergenerational panel discussion tackling taboo subjects such as mental health and wellbeing.

#### 4.2.5 Grandma's House Stakeholder Event

Our in‐person evaluation event brought together our team with community Elders who had contributed to our events, student intern and volunteer, local primary health care provider representative from Nottingham Deep End GP Research cluster, young people from Beechdale Community Centre Dads and Lads group and the retreat, service users and providers of the local African‐Caribbean day care centre for older people with dementia, physical or sensory issues.

Elders reflected positively on the opportunity to meet face to face, valuing the chance to speak openly with one another. Following a short presentation on the activities to date delivered by Grandma's House, the day centred around exploratory sessions. First, reflecting on the strengths and weaknesses of Grandma's House and secondly on the future opportunities and threats (SWOT).

### 4.3 Feedback and Learning

#### 4.3.1 Stephen Lawrence Day at Fernwood School, Nottingham

Discussions focused on experiences of school life, growing up, and wider society and culture.

Key differences between elders' and students' experiences emerged:

- Elders spoke about strict discipline, including corporal punishment, ‘fear, beating, changed how students acted’ and gendered subjects where girls were expected to do sewing and home economics but not gardening. They described learning respect through storytelling, being expected to care for younger children and older relatives, and experiencing a different standard of living in the Caribbean, such as no central heating and washing clothes in rivers. Many also recalled facing racism and slurs when growing up or settling in the United Kingdom.
- Students, in contrast, highlighted pressures such as expectations around crime, peer influence, especially through social media, and mental health challenges. They also noted that their school promotes inclusivity and encourages embracing different cultures.

The school praised our approach and indicated they would welcome further collaboration. We have since been approached by other local schools for our input.

#### 4.3.2 International Children's Day at the Pilgrim Church

Attendees described food as a powerful bond that brings families together, with grandmothers and elders seen as loving role models and guardians of tradition. The event created a safe, welcoming space where people felt seen, heard, and connected—across generations—through cooking, dialect, stories, and shared memories. Alongside this warmth and nostalgia, participants voiced concerns about young people's values, aspirations, and cultural education, and called for more regular intergenerational activities where parents, children, and elders come together to pass on traditions, guidance, and hope.

#### 4.3.3 Windrush Celebration at Beechdale Community Centre, Nottingham

Feedback highlighted deep reflections on health disparities, persistent yet subtle racism, and the erosion of cultural traditions and community bonds. Attendees shared memories of migration and early hardships, contrasted with concerns about generational disconnect and the fading respect for elders. Issues around healthcare bias, education, and inclusion were raised, alongside calls for teachers and professionals to better understand the needs of Black individuals. Nostalgia for strong family structures and communal life was evident, coupled with practical suggestions for rebuilding intergenerational connections through shared activities. Overall, the themes reveal a mix of pride in heritage, anxiety about cultural loss, and a desire for renewed unity and recognition.

Attendees spoke about how watching the play and then having time afterwards to reflect bought up memories of their past experiences with racism and prejudice; this included that faced by themselves, family members and friends. Attendees also fed back that the catering at the event brought back memories of food in Jamaica, prompting them to share stories with each other of food that they grew up with. The stalls around skincare and skin cancer in darker skin was also well‐received, with attendees feeling more educated and aware of how different skin conditions can present on darker skin tones and also relayed their own experiences of needing to explain to healthcare professionals and teachers how to be aware of skin conditions on darker skin.

#### 4.3.4 Dads and Lads, and StrongHER Retreat

Growing up, Grandma's House was a happy place, somewhere you felt valued and cared for, regardless of what challenges you were facing. Even after a difficult day or a challenging moment, it was a place where you could leave feeling empowered, encouraged, and worthy. The retreat sought to recreate that sense of safety, connection, and emotional well‐being for fathers, male carers, and young people, providing opportunities for meaningful conversations, reflection, and relationship‐building.

Reflections from Elders highlighted the challenges of integrating different generations with groups tending to congregate with their known peers. The Elders found the role reversal activities particularly enlightening, acknowledging the generational changes in parenting and language.

#### 4.3.5 Grandma's House Stakeholder Event

There was broad support for continuing Grandma's House. Conversations flowed around how to expand Grandma's House to better represent and include men and boys in discussions. Particularly around the role of elder men in guiding and mentoring boys and in creating a space where men and boys feel safe to explore taboo topics around mental health, dementia and grief: a ‘Grandad's Shed’ to include psychological, practical and educational activities. There were discussions around establishing arts‐based activities, such as mosaics, art installations, and holding events outside of community areas, for example, on University premises. The summary SWOT analysis is presented in Table 3. Elders and community leaders present welcomed the inclusion of the daycare service users and staff, and also teenage representatives from the Dads and Lads group.

**Table 3:** SWOT review of Grandma's House after 12 months.

Strengths Strong intergenerational relationships Building trust between community and university. Rich cultural identity and heritage Active community engagement Growing awareness and openness around mental health Emerging creative project ideas Collective learning and knowledge sharing	Weaknesses Loss of past community cohesion Limited opportunities for older men Challenges in discussing sensitive topics Career advice and opportunities remain uneven Financial pressures influencing youth behaviour University processes inflexible and slow
Opportunities Intergenerational storytelling and heritage sharing Creative and therapeutic arts Expansion of Dads and Lads programme New community spaces Health awareness initiatives Dementia and grief support	Threats Mental health vulnerabilities Economic challenges Erosion of historical community structures Dementia and ageing challenges Ongoing stigma around seeking help

Overall, there was enthusiasm from all in attendance to continue Grandma's House and that it was breaking down barriers between the local African‐Caribbean community and the university.

### 4.4 Reflexivity Statement

The academic lead authors identify as White British women and recognise that their social, cultural and professional positioning may have shaped how this project was framed, interpreted and written. In research exploring experiences within African‐Caribbean communities, we acknowledge that our perspectives are informed by different lived experiences and histories, which may influence how data are understood and represented. We therefore recognise the potential for unconscious assumptions, blind spots and power imbalances within the research process.

To address these challenges, this paper was co‐produced and co‐authored with community partners of African‐Caribbean heritage, whose lived experience, cultural knowledge and insights informed all stages of the work, including study design, interpretation of findings and manuscript development. Their contributions were central to ensuring that community perspectives were accurately represented and that interpretations remained grounded in the realities of participants' experiences.

The project also benefited from the involvement of two student researchers from ethnically diverse backgrounds, whose perspectives contributed additional critical reflection and helped broaden discussions during analysis and interpretation. Through ongoing dialogue within the team, differing viewpoints were openly discussed and challenged, supporting a more nuanced understanding of the findings.

While co‐production and collaborative authorship cannot remove all power differentials or biases, these processes strengthened the credibility and cultural relevance of the work. We acknowledge that the interpretations presented remain shaped by the identities, experiences and positions of all members of the research team and should be understood within this context.

We also recognise that academic authors retain structural privilege within research institutions, publication processes and knowledge production. Co‐authorship with community partners was an intentional strategy to share interpretive authority, although we acknowledge that complete equity in these processes is difficult to achieve.

## 5 Discussion

### 5.1 Principal Findings

Our experience identifies opportunities to facilitate a critical shift in the role of community members in leading collaborations in health research and practice. Grandma's House worked because it began with cultural pride rather than community need. The idea came from within the community, and academics joined it, not the other way around. That shift matters. It communicates that lived experience, heritage and relational knowledge are forms of expertise, restoring dignity before seeking data.

Our findings contrast with many public engagement initiatives in which engagement methods originate from professional or institutional agendas, even when subsequently co‐developed with communities. Whilst we applaud all efforts to engage the public in research, we were keen to create a genuinely community‐led approach ‐ aligning with the upper levels of Arnstein's Ladder of Participation [24]. Building on existing positive social identities is not unique in public involvement in research [2, 25]. Primary learning about creating such a vision identified that engagement must feel like belonging, not recruitment; equity must be materially demonstrated; and that flexibility protects trust. Sharing food, stories and laughter created safety. Only then could conversations about sensitive topics such as racism, grief, mental health and identity surface honestly. We were reminded that meaningful involvement grows from relationships. When people feel that they belong in a space, research dialogue becomes possible. Without belonging, involvement becomes transactional.

Two interconnected mechanisms appeared central to the success of Grandma's House: (i) cultural heritage and belonging, and (ii) trust and trustworthiness.

### 5.2 Interpreting the Findings

#### 5.2.1 Cultural Heritage and Belonging

From the community's perspective, protecting cultural heritage is a public health intervention. It strengthens social cohesion, identity formation and intergenerational trust, all of which underpin healthy families. Grandma's House recreated experiences many families value. It offered proximity to Elders, shared meals, dialect, storytelling and informal advice. From a public health perspective, that reduces social isolation and strengthens protective social factors.

The importance of cultural heritage within Grandma's House can be interpreted through Gadamer's [19] concept of historically situated understanding. Participants did not enter the space as isolated individuals but as members of communities carrying shared histories, traditions and experiences. Storytelling, dialect, food and intergenerational exchange may have facilitated participation through recognition of these shared horizons of understanding. From this perspective, engagement was not achieved simply by removing practical barriers but by creating opportunities for researchers and community members to enter a dialogical process in which different forms of knowledge could be negotiated and understood.

Cultural heritage functioned not only as the foundation for engagement but also to signal trustworthiness. Community leadership, cultural familiarity and shared identity created conditions in which people felt recognised, respected and belonged.

#### 5.2.2 Trust and Trustworthiness

Trust emerged as a foundational condition for meaningful public engagement. Trust is particularly important in public‐facing research and policy settings, where individuals and communities engage with institutions under conditions of uncertainty. Luhmann [26] conceptualises trust as a mechanism through which uncertainty is managed. This perspective helps explain why community members may initially approach research organisations with caution. For communities that have experienced exclusion, discrimination or extractive forms of engagement, uncertainty regarding institutional motives is neither hypothetical nor unfounded. In this context, mistrust should not be viewed as a deficit to be overcome, but as a rational and protective response to previous experiences. Responsibility, therefore, lies less with communities to become trusting and more with institutions to demonstrate sustained trustworthiness through transparency, accountability and meaningful power‐sharing [27].

From this perspective, the success of Grandma's House can be understood not as the achievement of trust itself, but as the creation of conditions in which trustworthiness could be demonstrated. Food, storytelling, informal conversation, repeated interactions and visible community leadership reduced uncertainty and facilitated reciprocal relationship‐building. These activities communicated respect for community knowledge and cultural heritage, moving beyond instrumental models of engagement in which participation is sought primarily to meet organisational objectives. Instead, trust developed through consistent interactions that recognised lived experience, cultural identity and relational knowledge as valuable forms of expertise.

However, trust remained contingent and fragile. Social trust is built slowly through reciprocal interactions but can be eroded rapidly through perceived inconsistency or disregard for community priorities [28, 29]. Luhmann [26] argues that trust cannot be demanded or restored through reassurance alone; it must be grounded in demonstrable trustworthiness. Maintaining trust, therefore, requires more than positive initial engagement, highlighting the importance of ongoing responsiveness, accountability and ethical reflexivity. Without such efforts, engagement risks becoming performative or extractive, ultimately undermining legitimacy and long‐term public confidence.

### 5.3 Challenges and Tensions

#### 5.3.1 Working Within Conventional Research Structures

Sustaining trust can be difficult when community‐led approaches operate within conventional research structures [30]. Inflexibility in research organisation processes emerged as a significant barrier. Standardised administrative procedures, rigid timelines and narrowly defined deliverables often conflicted with the relational and adaptive nature of community‐based work. Such constraints risk signalling that institutional priorities remain privileged over community priorities, thereby reinforcing existing power asymmetries. Viewed through a Luhmannian lens [26], these organisational practices can reintroduce uncertainty about institutional intentions and threaten the trust that engagement activities seek to establish. Financial pressures and short‐term funding models further compound this challenge by prioritising measurable outputs over the long‐term relationship‐building necessary for genuine co‐production.

#### 5.3.2 Reflecting Diverse Community Perspectives

While Grandma's House emerged from a community‐led vision, it is important to recognise that communities are not homogeneous entities. A Gadamerian [19] perspective reminds us that no single horizon represents an entire community. Community priorities, identities and experiences are often diverse and sometimes conflicting. The voices most visible within community‐led initiatives may not necessarily reflect those of all community members, particularly younger people, more recent migrants, or those less connected to existing social networks. The absence of substantial documented feedback from younger attendees suggests that some perspectives remained less visible within the dialogue than others.

These challenges may be exacerbated by wider socioeconomic changes that weaken community cohesion and reduce collective capacity for participation. Engagement may therefore become concentrated among a relatively small number of already‐engaged individuals. Future work should remain attentive to whose perspectives are represented, whose may continue to be marginalised despite community‐led intentions and how it can support the rebuilding of social connections and continuity.

### 5.4 Implications for Research and Practice

Using Arnstein's Ladder of Participation [24], Grandma's House can be understood as operating between partnership and delegated power rather than full citizen control. The original concept emerged from within the African‐Caribbean community, and community partners played a central role in shaping priorities, event design and delivery. However, funding, governance and reporting requirements remained linked to university and funder structures. This illustrates a persistent tension identified by Arnstein, whereby influence may be redistributed whilst ultimate authority remains embedded within institutional systems. The strength of the initiative, therefore, lay not in a complete transfer of power but in a meaningful shift of agenda‐setting from academic to community partners.

As noted above, community‐engagement initiatives are rarely agenda‐free. Researchers, funders, and healthcare organisations typically enter such partnerships with specific goals, including improving research participation, reducing inequalities, or informing service development. The experience of Grandma's House suggests that community members were receptive not because external agendas were absent, but because these agendas were secondary to the development of authentic relationships, reciprocal benefit, and opportunities for communities to shape engagement activities themselves.

The ultimate aim of Grandma's House is to improve research inclusion by using trust as a mediating mechanism. Drawing on principles of community‐engaged research and community‐based participatory research, we propose that community engagement contributes to increased participation in clinical trials indirectly through a series of intermediate mechanisms. These include the development of trust, increased awareness and understanding of research, improved alignment of research with community priorities, and stronger relationships between communities and research institutions. Together, these mechanisms are hypothesised to increase both the willingness and opportunity of underserved populations to participate in clinical research, ultimately contributing to more equitable clinical trial participation (Theory of Change—Supporting Information S1: Figure 1).

While cultural heritage provided a powerful foundation for engagement, there is a risk that culturally grounded interventions can be interpreted as sufficient responses to health inequities. Such approaches should not obscure the structural factors that shape health, including racism, economic inequality, housing conditions, and differential access to services. Cultural assets can strengthen resilience and belonging, but they cannot substitute for action on the wider social determinants of health.

There is also a risk that successful community‐led initiatives become incorporated into institutional narratives of engagement without corresponding shifts in power or resource allocation. Community organisations may be expected to provide cultural legitimacy, trust and access while remaining dependent upon short‐term funding streams controlled by academic institutions. Sustained commitment to community leadership, therefore, requires redistribution of resources as well as recognition of community expertise.

### 5.5 Strengths and Limitations

A major strength of this project was its community‐led nature. It was initiated from an idea voiced by M.T., a community advocate, and subsequently led by M.T. and community partners once funding was secured. This enabled community priorities, cultural heritage and local knowledge to shape both the purpose and delivery of the initiative.

A further strength was the use of flexible, inclusive and unobtrusive methods for capturing attendees' feedback and reflections. However, this also represents a limitation as feedback consisted largely of short, ad hoc comments rather than data captured through a comprehensive evaluation framework.

As previously mentioned, this initiative may also have under‐represented some community perspectives, particularly those of younger attendees and individuals less connected to established social networks.

This paper is reported in line with GRIPP2 guidance, ensuring clear reporting of patient and public involvement throughout (GRIPP2‐SF, Supporting Information S1: Table S1).

## 6 Conclusion

We have demonstrated that community‐led, culturally grounded approaches such as Grandma's House are not ancillary to health research and practice, but foundational to their legitimacy and effectiveness. By beginning with cultural pride, belonging, and relational knowledge, rather than deficit or need, Grandma's House repositions communities as originators of insight and action, and researchers as partners rather than drivers.

The model illustrates how cultural heritage can function simultaneously as a mechanism for trust‐building, a public health intervention, and a platform for authentic co‐production. However, its success also exposes the limitations of prevailing research systems that privilege inflexibility, short‐term funding, and predefined outputs over relational work. If research institutions are serious about equity and sustained public engagement, they must adapt structures, funding models, and success metrics to support community leadership, long‐term trust maintenance, and the rebuilding of social cohesion. Without such systemic change, the burden of engagement innovation will continue to fall disproportionately on communities themselves, while the transformative potential of truly inclusive research remains unrealised.

More fundamentally, this work raises questions about who defines research priorities, whose knowledge is recognised as legitimate, and who benefits from engagement activities. Community‐led approaches challenge longstanding assumptions that expertise resides primarily within academic institutions. However, unless research systems are willing to relinquish aspects of control and redistribute decision‐making authority, community leadership may remain more aspirational than realised.

## Author Contributions

**Adele Horobin:** conceptualisation, methodology, data curation, investigation, validation, formal analysis, funding acquisition, project administration, writing – original draft, writing – review and editing. **Monica Tucker:** conceptualisation, methodology, writing – review and editing, investigation. **Shanine Fasasi:** methodology, investigation, writing – review and editing. **Gemma Poole:** investigation, methodology, writing – review and editing. **Khadiyo Hassan:** investigation, formal analysis, writing – review and editing. **Insa Hussain:** investigation, writing – review and editing, formal analysis. **Joanne R. Morling:** methodology, data curation, investigation, validation, formal analysis, supervision, funding acquisition, project administration, writing – original draft, writing – review and editing.

## Ethics Statement

As this article reports on the development and delivery of an approach, it was not considered research and no formal ethical approval was obtained. At all stages of the process, people involved were fully informed of the developmental nature of the project and the intention to share learnings.

## Conflicts of Interest

The authors declare no conflicts of interest.

## Supporting information


Supporting File

## Data Availability

This is an article reporting on the development and delivery of an approach. No formal research was undertaken, and no formal data is available.

## References

[1] Z. Gray , “INVOLVE: Supporting Public Involvement in NHS, Public Health and Social Care Research” [Internet], 2025, https://www.nhsresearchscotland.org.uk/uploads/tinymce/Zoe%20Gray.pdf.

[2] L. Holmes , K. Cresswell , S. Williams , et al., “Innovating Public Engagement and Patient Involvement Through Strategic Collaboration and Practice,” Research Involvement and Engagement 5, no. 1 (October 2019): 30, 10.1186/s40900-019-0160-4. PMC6802177 31646001

[3] Partnership for Black People's Health , “Centering Black Voices in Health Equity” [Internet], 2025, https://www.qmul.ac.uk/wiph/centres/centre-for-public-health-and-policy/global-public-health-unit/p4bph/.

[4] Z. Ersoy Guller , F. Green , and A. L. Goodman , “Underrepresentation of Ethnic Minorities in UK COVID‐19 Trials: Comment on a Recent Systematic Review and Meta‐Analysis,” BMC Medicine 21, no. 1 (August 2023): 308, 10.1186/s12916-023-03002-6. PMC10426162 37580724

[5] I. B. Riaz , M. Islam , W. Ikram , et al., “Disparities in the Inclusion of Racial and Ethnic Minority Groups and Older Adults in Prostate Cancer Clinical Trials: A Meta‐Analysis,” JAMA Oncology 9, no. 2 (February 2023): 180–187, 10.1001/jamaoncol.2022.5511. PMC9685549 36416812

[6] M. Canevelli , G. Bruno , G. Grande , et al., “Race Reporting and Disparities in Clinical Trials on Alzheimer's Disease: A Systematic Review,” Neuroscience & Biobehavioral Reviews 101 (June 2019): 122–128, 10.1016/j.neubiorev.2019.03.020. 30946856

[7] “Taking Stock—NIHR Public Involvement and Engagement,” NIHR [Internet], accessed June 13, 2025, https://www.nihr.ac.uk/taking-stock-nihr-public-involvement-and-engagement.

[8] A. Hunn , “Survey of the General Public: Attitudes Towards Health Research 2017” [Internet], Health Research Authority (HRA) and National Institute for Health Research (NIHR), 2017, chrome‐extension://efaidnbmnnnibpcajpcglclefindmkaj/, https://s3.eu-west-2.amazonaws.com/www.hra.nhs.uk/media/documents/HRA_NIHR_general_public_omnibus_survey_2017_FINAL.pdf.

[9] P. Bower , C. Grigoroglou , L. Anselmi , et al., “Is Health Research Undertaken Where the Burden of Disease Is Greatest? Observational Study of Geographical Inequalities in Recruitment to Research in England 2013–2018,” BMC Medicine 18, no. 1 (May 2020): 133, 10.1186/s12916-020-01555-4. PMC7232839 32418543

[10] R. D. Putnam , “Bowling Alone: The Collapse and Revival of American Community (Simon and Schuster, 2000).

[11] P. Bourdieu , “The Forms of Capital (1986),” Cultural Theory: An Anthology 1, no. 81–93 (2011): 949.

[12] J. S. Coleman , Foundations of Social Theory (Harvard University Press, 1990).

[13] M. Woolcock , “Social Capital and Economic Development: Toward a Theoretical Synthesis and Policy Framework,” Theory and Society 27, no. 2 (1998): 151–208.

[14] D. Cooperrider and S. Srivastva , “Appreciative Inquiry in Organizational Life,” in Appreciative Inquiry: Foundations in Positive Organization Development, eds. D. Cooperrider , L. Sorensen , P. Yager , and T. Whitney (Champaign Ill Stipes Publ, 2005), 104.

[15] J. P. Kretzmann and J. McKnight , Building Communities From the Inside Out: A Path Toward Finding and Mobilizing a Community's Assets, (Acta Publications, 1993).

[16] R. J. Lewicki and B. B. Bunker , “Developing and Maintaining Trust in Work Relationships,” in Trust in Organizations: Frontiers of Theory and Research, ed. R. M. Kramer and T. R. Tyler (SAGE Publications, 1996), 114–139.

[17] P. Blau , Exchange and Power in Social Life (Routledge, 2017).

[18] C. Huxham and S. Vangen , Managing to Collaborate: The Theory and Practice of Collaborative Advantage (Routledge, 2013).

[19] K. Dockhorn and M. Brown , “Hans‐Georg Gadamer's Truth and Method,” Philosophy & Rhetoric 13, no. 3 (1980): 160–180.

[20] J. Ocloo , S. Garfield , B. D. Franklin , and S. Dawson , “Exploring the Theory, Barriers and Enablers for Patient and Public Involvement Across Health, Social Care and Patient Safety: A Systematic Review of Reviews,” Health Research Policy and Systems 19, no. 1 (2021): 8. 10.1186/s12961-020-00644-3 PMC7816359 33472647

[21] M. Dreyer , H. Kosow , A. Bauer , B. Chonkova , V. Kozarev , and L. Timotijevic , “Public Engagement With Research: Citizens' Views on Motivations, Barriers and Support,” Research for All 5, no. 2 (September 2021): 302–319, 10.14324/RFA.05.2.08.

[22] D. Snape , J. Kirkham , J. Preston , et al., “Exploring Areas of Consensus and Conflict Around Values Underpinning Public Involvement in Health and Social Care Research: A Modified Delphi Study,” BMJ Open 4 (January 2014): e004217, 10.1136/bmjopen-2013-004217. PMC3902382 24413356

[23] J. M. Watkins , B. J. Mohr , and R. Kelly , Appreciative Inquiry: Change at the Speed of Imagination (John Wiley & Sons, 2011).

[24] S. R. Arnstein , “A Ladder of Citizen Participation,” Journal of the American Institute of Planners 35, no. 4 (July 1969): 216–224, 10.1080/01944366908977225.

[25] S. Islam , O. Joseph , A. Chaudry , et al., “We Are Not Hard to Reach, But We May Find It Hard to Trust”. Involving and Engaging ‘Seldom Listened To’ Community Voices in Clinical Translational Health Research: A Social Innovation Approach,” Research Involvement and Engagement 7, no. 1 (June 2021): 46, 10.1186/s40900-021-00292-z. PMC8234650 34174961

[26] N. Luhmann , Trust and Power (John Wiley and Sons, 1979).

[27] S. Camporesi , M. Vaccarella , and M. Davis , “Investigating Public Trust in Expert Knowledge: Narrative, Ethics, and Engagement,” Journal of bioethical inquiry 14, no. 1 (2017): 23–30. 10.1007/s11673-016-9767-4 PMC5340832 28144901

[28] L. Gilson , “Trust and the Development of Health Care as a Social Institution,” Social Science & Medicine 56, no. 7 (2003): 1453–1468. 10.1016/s0277-9536(02)00142-9 12614697

[29] J. Doyle , “Fragile Trust, Stable Mistrust: A Theory of the Formation and Sustainability of Social Trust,” Social Psychology Quarterly 87, no. 4 (2024 Dec 1): 333–356, 10.1177/01902725231204848.

[30] B. G. Robbins , “From the General to the Specific: How Social Trust Motivates Relational Trust,” Social Science Research 55 (2016): 16–30. 10.1016/j.ssresearch.2015.09.004 26680285

