# Supplementary material for: Grandma's House: A Community‐Led Partnership With University Researchers Drawing on Cultural Heritage Approaches to Support Research Development
Source: Health Expect. 2026 Oct 27;29(5):e70932. doi: 10.1111/hex.70932 (PMC13711016; doi:10.1111/hex.70932)
Supplement: Supplementary file 1 [file HEX-29-e70932-s001.docx]

**Supplementary Figure S1.**

**Theory of Change for Grandma’s House.**

**Supplementary Table S1.**

**GRIPP2-SF**

| **Section and topic** | **Item** | **Reported on page No** |
| --- | --- | --- |
| **1: Aim** | **Report the aim of PPI in the study:**  Abstract; Community members were originators and drivers for the initiative and Aims & Objectives re: Community originators and champions and the aim to establish a community-led, shared initiative. | Page 1, 4. |
| **2: Methods** | **Provide a clear description of the methods used for PPI in the study**:  Method used was co-production through a leadership team of community members and university members, with decisions made by consensus according core principles of community first.  Individual strengths acknowledged and it was exemplified where community leaders led (defining the initiative, proposing and developing activities, who to involve and how and who to promote to, venues to use, event timings) and where university staff led (facilitating, co-ordinating, identifying, obtaining and administering funds) | Page 5 |
| **3: Study results** | **Outcomes—Report the results of PPI in the study, including both positive and negative outcomes:**  Reported that our community partners shaped the vision of Grandma’s House as replicating the generation-to-generation support model centred on the role of the Grandmother as matriarch., with associated objectives to strengthen the community, create opportunities to build relationships with researchers and services, tackle taboos, and to do this through events to connect generations.  We reported on the events co-delivered and which communities were involved in these, and the learning and feedback we gained from these events.  We also included a Reflexivity statement to acknowledge authors’ positioning and how we used coproduction and collaborative authorship to address power differentials and strengthen the credibility and cultural relevance of the work | Page 7, 8, 9, 10, 11, 12 |
| **4: Discussion and conclusions** | **Outcomes—Comment on the extent to which PPI influenced the study overall. Describe positive and negative effects:**  We acknowledged in our reflexivity statement that coproduction and collaborative authorship cannot remove all power differentials and biases and that academic authors retain structural privilege within research institutions, publication processes and knowledge production.  We discussed the placement of our community-led approach on the upper rungs of the Arnstein’s ladder of participation.  We highlighted how being community-led ensured a culturally-informed approach and engagement practice steeped in cultural heritage.  Also, that the visibility of the community led approach to community members was an essential part of projecting trustworthiness. | Page 17-22 |
| **5: Reflections/critical perspective** | **Comment critically on the study, reflecting on the things that went well and those that did not, so others can learn from this experience**  We recognised that communities are diverse and some community voices may have been more heard than others in our community-led initiatives.  We highlighted the challenges that rigid, process-driven mechanisms within academic research structures pose to our co-production and community-led approach.  In lessons for future practice, we highlighted that:   - There is a risk that culturally grounded interventions can be interpreted as sufficient responses to health inequities. - There is a risk that successful community-led initiatives become incorporated into institutional narratives of engagement without corresponding shifts in power or resource allocation.   We cautioned that culturally-informed practice is not a substitute for action on the wider determinants of health; also that commitment to community leadership requires redistribution of resources. | Page 19, 20, 21, 22 |
